# The use of artificial neural network for low latency of fault detection and localisation in transmission line

**DOI:** 10.1016/j.heliyon.2023.e13376

**Published:** 2023-02-02

**Authors:** Vincent Nsed Ogar, Sajjad Hussain, Kelum A.A. Gamage

**Affiliations:** Department of Electrical and Electronic Engineering, James Watt School of Engineering, University of Glasgow, Glasgow, United Kingdom

**Keywords:** Transmission line, Fault protection, Machine learning, Fault detection, Fault localisation

## Abstract

One of the most critical concerns in power system reliability is the timely and accurate detection of transmission line faults. Therefore, accurate detection and localisation of these faults are necessary to avert system collapse. This paper focuses on using Artificial Neural Networks in faults detection and localisation to attain accuracy, precision and speed of execution. A 330 kV, 500 km three-phase transmission line was modelled to extract faulty current and voltage data from the line. The Artificial Neural Network technique was used to train this data, and an accuracy of 100% was attained for fault detection and about 99.5% for fault localisation at different distances with 0.0017 μs of detection and an average error of 0%–0.5%. This model performs better than Support Vector Machine and Principal Component Analysis with a higher fault detection time. This proposed model serves as the basis for transmission line fault protection and management system.

## 1 Introduction

The electrical power system is comprised of interconnected parts such as power generation, transmission, and distribution. The transmission line is an essential component of the power system since it transports energy from the producing plant to the end customers. These components are linked via transmission lines, which are prone to malfunction and can only be managed remotely through complex processes [1]. The transmission line's difficulties are exacerbated by ageing gear, lightning, human contact, and extreme weather conditions. However, power quality is the most critical component in an electrical network. When a transmission line fails, the electricity quality drops, which directly impacts power output [2].

Fault detection and localisation are critical for safeguarding a transmission line's network. As a result, proper precautions must be taken to provide optimum protection and avoid system failure. The fault must be identified to prevent the transmission line from damage, and the fault location must be precise for speedy line isolation [3]. However, fault detection input may considerably aid problem localisation for faster fault clearing and power restoration [4]. Identifying the location of a transmission line failure in a power system is crucial for rapid response and power supply dependability [5].

Transmission line identification and localisation have mostly been performed using classic machine learning and deep learning approaches. As a switching mechanism for fault protection, the typical way employs a distance protection relay over current and voltage relays [6,7]. Mobile robots [[8], [9], [10], [11]] are used to identify line problems and monitor transmission lines. Furthermore, the fuzzy logic technique [12,13,[13], [13], [14], [15]] Neuro-fuzzy method [14,16] wavelet and fuzzy approach [12,17] wavelet and fuzzy approach While the Artificial Neural Network (ANN) is a component of the machine learning technique [[18], [19], [20], [21], [22]], the Support Vector Machine (SVM) [1,23,24], and decision tree (DT) [28] are also used. When time and frequency data are needed, the WT is helpful but vulnerable to noise and harmonics. Therefore, it has certain limits. The method of producing a reference wavelet is time-consuming and involves a high sampling rate. Furthermore, the number of decompositions is generated via experiments and is mainly used for defect detection [26]. Back-propagation neural networks were employed as an alternate defect detection and localisation approach in Ref. [27]. This may be used to create a long transmission line distance relay protection mechanism. However, the model's fault classification accuracy is weak.

Many hybrid approaches, such as the S-transform and ANN, have been used to improve performance in fault localisation and detection [28]. This approach was used to discover transmission line faults despite the fact that it did not take into consideration a multi-class data set of fault data [28]. Furthermore, the ANN and SVM were used in the defect detection process, and since they demand a large quantity of data for training, they are difficult to maintain and take a long time. WT is also used in fault detection. However, distinguishing between various fault states is difficult [29]. Even though the bulk of these strategies is very new, there are a number of challenges. The high processing cost and the unsuitability of the Hilbert-Huang Transform (HHT) for high-frequency signals, as seen in Ref. [30].

Principal Component Analysis is a rapid and easy machine learning approach that reduces re-projection error and is noise-resistant (PCA). However, if the number of dimensions exceeds the number of data points, the convergence matrix will always be enormous, making it impossible to get [31]. The PCA is also used to map data from high dimensional space to low dimensional subspace to decrease the data's dimensionality and better understand its variance. According to studies, by training their algorithms on smaller datasets, the bulk of these methodologies provide fairly accurate results. Furthermore, for training, they use data from either a single phase to ground fault or a double phase to line fault [[32], [33], [34]]. Single Phase, Double Phase, and Three Phase to Ground Fault datasets will be used concurrently to identify and localise the transmission line fault, with a focus on machine learning and deep learning approaches.

DWT and DT [35] have poor performance for high-performance faults and limited temporal resolution capabilities when considering fault location. Using data mining and wavelets [36], Decision Tree (DT) and K-Nearest Neighbors (KNN) are used, although they do not quantify fault location [37]. The S-transform approach was the only one investigated in Ref. [38]. For fault identification and detection, morphology in mathematics and the Recursive Least-square (RLS) method [39] are employed to identify fault characteristics based on mathematical morphology. This approach is difficult to maintain since it is employed in incredibly intricate situations that are not home in nature. Another disadvantage of the previously discussed approaches was their inability to focus more on fault localisation. Localisation of faults facilitates rapid diagnosis and power restoration during an outage by pinpointing the exact location of the problem.

Transmission line fault analysis normally requires three main activities for a successful fault management system: fault sensing or detection, categorising the problem into various categories and identifying the spot to disclose the zone where the fault occurred [3]. The extraction of fault features is being considered. This may be accomplished by first modelling the network in MATLAB/SIMULINK to extract fault instances from the transmission line. The next step is to identify and localise the flaws using data provided by the simulated model and an ANN-trained classifier [39].

Because of the delay in fault detection and the increased role of communication and computers in transmission systems [40], this study aims to offer a novel ANN-based approach for rapid, reliable, and accurate fault identification and localisation in transmission lines. Also, to detect many fault circumstances, such as defective voltage and current, simultaneously minimise fault detection time delay. The suggested algorithm's performance was assessed by simulating several errors and training them with the ANN model, and the results were encouraging. In addition, the suggested model will be used to develop transmission line fault management and protection in power systems.

One of the technique's significant disadvantages is the model's inability to train on non-numerical data. Therefore, interpreting the findings is always challenging as matching results with real-life circumstances and issue statements.

## 2 The Artificial Neural Network technique

Artificial Neural Networks have traditionally been used with great success in various sectors of fault analysis. This is one of the most extensively utilised artificial intelligence technologies, which is critical in constructing a strong power system failure management model. An ANN model typically has three major layers: input, hidden, and output. The input layer receives data or signals from the model, which can be fault current or voltage sent into the model. The hidden layer extracts patterns associated with the analyzed process or system. The output is in charge of creating and displaying the final network output. These layers handle most internal network processing, which involves the results processed from other layers. ANN has several advantages that allow it to be widely used in developing fault management models that are exceptionally effective [41].

**Fig. 1:**
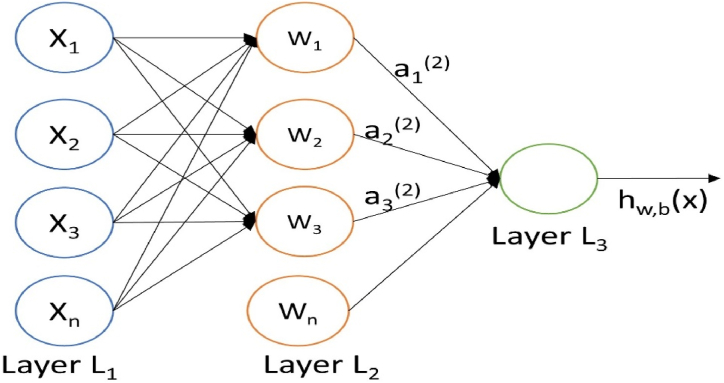
Artificial neural network model.

Fig. 1 is a multi-layer neural network, and *X*_*1*_*, X*_*2*_*, …, X*_*n*_ represents the external source input signal which represents the faulty current and voltage signals. *W*_*1*_*, W*_*2*_*, …, W*_*n*_ represent every input variable's synaptic weights and enable the evaluation of their importance to the model's functionality. For a fixed training dataset of $\left(x^{\left(1\right)},y^{\left(1\right)}\right)$, …, $\left(x^{\left(m\right)},y^{\left(m\right)}\right)$ of m training sample, and using the batch gradient decent, the cost function for a single dataset is given in equation (1) below.

(1) $$J\left(W,b;y\right)=\frac{1}{2}\;\left|\left|,b\left(x\right)-y\right|\right|$$

While, for a training set of m sample is defined in equation (2)

(2) $$J\left(W,b\right)=\left[\frac{1}{m}\;\sum_{i=1}^{m}J(W,b;x^{i},y^{i})\right]+\frac{\lambda }{2}\sum_{l=1}^{nl-1}\sum_{i=1}^{s}l\;\sum_{j=1}^{sl-1}{\left(W_{ji}^{\left(l\right)}\right)}^{2}$$

(3) $$=\left[\frac{1}{m}\sum_{i=1}^{m}\left(\frac{1}{2}∥hw,b{\left(x\right)}^{\left(i\right)}\right)-y^{\left(i\right)}{∥}^{2}\right]+\frac{\lambda }{2}\sum_{l=1}^{nl-1}\sum_{i=1}^{s}l\;\sum_{j=1}^{sl-1}{\left(W_{ji}^{\left(l\right)}\right)}^{2}$$

Where J(W,b) in equation (2) is an average sum of square errorterm, and equation (3) is the weight decay or regularisation term that decreases the magnitude of the weight, and helps to prevent overfitting. Also, λ is the weight decay parameter. Σ is the linear aggregator that combines all input signals weighted by synaptic weights to generate an activation voltage. θ Is the activation threshold or bias used to identify the suitable point that the linear aggregator makes. U denotes the activation potential. If this value is positive, then *u ≥* θ, the model has an excitatory potential; otherwise, it will be inhibitory. g is the activation function, while y is the output signal.

The result produced by ANN, as proposed by McCulloch and Pitts [40], is represented in equation (4) below.

(4) $$u=\sum_{i=1}^{n}w_{i}.x_{i-\theta }$$

And y=g(u). Therefore, the ANN gives the model a set of values that reflect the input variable by multiplying each neuron input from its associated synaptic weight, calculating the activation potential from the weighted sum of the input signal, and removing the activation threshold. It also employs an appropriate activation function in the activation potential to restrict neuron output and assemble the output by applying the neural activation function.

The error of a neuron j in the output layer Y is given in equation (5) below.

(5) $$E_{j=}\frac{1}{2}\;{\left(R_{j}-Y_{j}\right)}^{2}$$

Where $R_{j}$ is the predetermined value, and the total error E of the output layer is shown in equation (6)

(6) $$E=\sum_{j}E_{j}=\frac{1}{2}\;\sum_{j}{\left(R_{j}-Y_{j}\right)}^{2}$$

To reduce the error E in terms of the weight change $\Delta W_{kj}$ and using the delta rule to integrate the learning rate α in addition to the gradient descent method strategies for defining weight changeas shown in equation (7)

(7) $$\Delta W_{kj}=-\alpha .\frac{\partial E}{\partial W_{kj}}\;;0<\;\alpha \leq 1$$

The weight change will be negative if the gradient is positive and vice versa if the gradient is negative. As a result, a negative symbol has been placed on the right side. It has been demonstrated that backpropagation learning with enough hidden layers may estimate any nonlinear function to arbitrary precision. As a result, back propagation learning neural networks are excellent for signal prediction and system modelling [27].

**Fig. 2:**
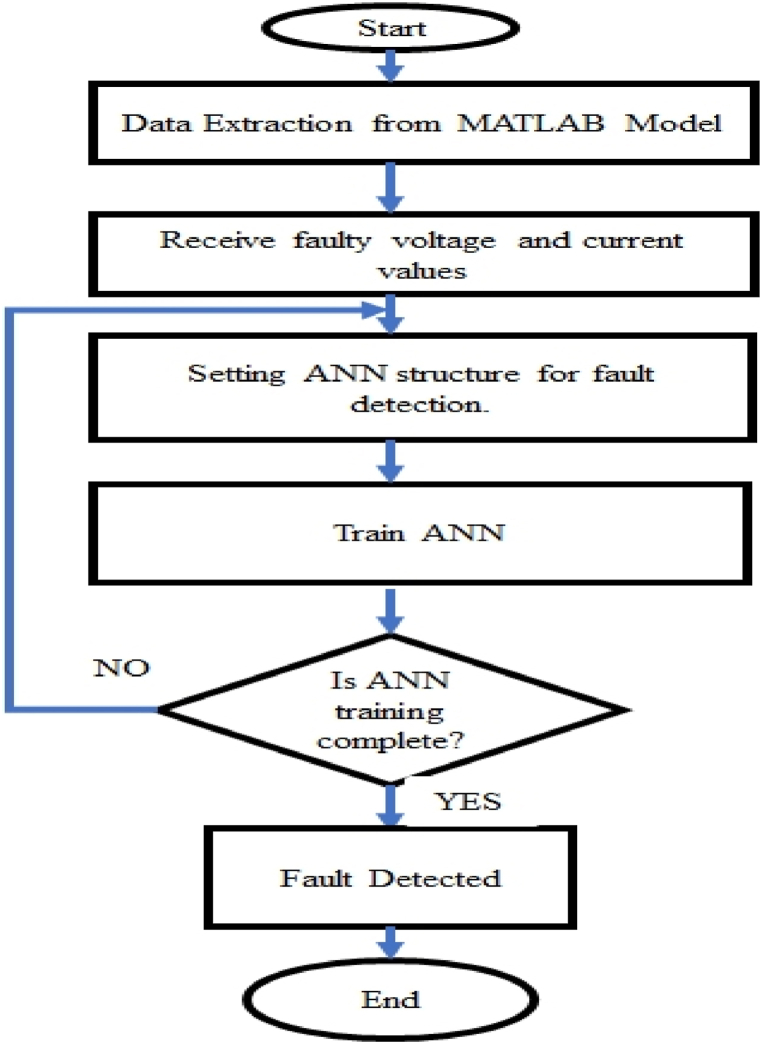
Flow chart for Proposed algorithm.

The flow chart in Fig. 2 describes how the algorithm is executed from the data extraction to fault detection. This is achieved by.

i. Presenting a set of values to the neuron, representing the input variables.
ii. Each neuron's input should be multiplied by the synaptic weight that corresponds to it.
iii. The activation potential created by the weighted sum of the input signals is obtained, and the activation threshold is subtracted.
iv. Using a suitable activation function to reduce the output of the neurons.
v. Utilise the neural activation function in the activation potential to compile the output

The multiple layers, which are made up of one or more hidden neural layers, are used to solve a variety of issues, such as those involving function approximation, pattern classification, system identification, process control, optimisation and robotics [41] The multi-layer typically has three classes of layers: an input layer, which is used to transfer the input vector to the network, hidden layers of computation neurons, and an output layer, which is made up of at least one computation neuron and produces the output vector [42].

## 3 Modeling of a 330 kV transmission line

**Fig. 3:**
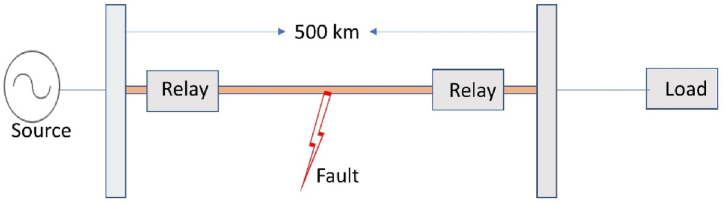
A 330 kV three-phase, 500 km transmission line model.

The Artificial Neural Network technique needs many datasets for practical training, and those datasets are obtained from the model in Fig. 3 below.

**Fig. 4:**
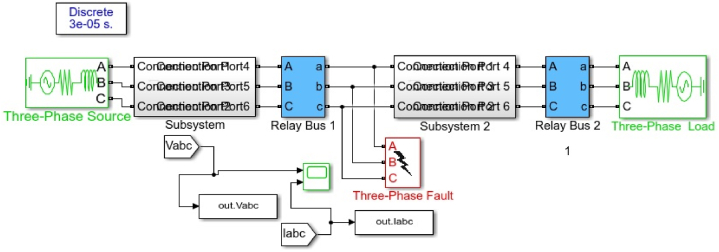
Simulink Model of 330 kV, 500 km Transmission line.

The parameters from Table 1, Table 2 are used to model the Simulink in Fig. 4. This model generates fault data of single line to ground, double line to ground and three-phase to ground fault. This data is used to train the machine learning using ANN algorithm to check for fault detection, classification and localisation. It will also be used to validate the data for accuracy, Root Mean Square Error (RMSE) and precision of result location.

**Table 1:** The parameters of 330 kV, 500 km transmission line.

Sequence	parameter	Value	Unit
Positive and negative sequence resistance	R_1_, R_2_	0.01273	Ω/km
Zero sequence resistance	R_0_	0.3864	Ω/km
Positive and negative sequence inductance	L_1_, L_2_, L_3_	0.9337 × 10^−3^	H/km
Zero sequence inductance	L_0_	4.1264 × 10^−3^	H/km
Positive and negative sequence capacitance	C_1_, C_2_, C_3_	12.74 × 10^−9^	F/km
Zero sequence capacitance	C_0_	7.751 × 10^−9^	F/km

**Table 2:** Fault parameters of the proposed model.

**System components**	**Parameters/units**	**Value**
**Phase to phase voltage**	voltage	330
**Source resistance Rs**	Ohms (Ω)	0.8929
**Source inductance**	H	16.58 × 10^−3^
**Fault incipient angle**	θ in degree	0° and −30°
**Fault resistance Ron**	Ohms (Ω)	0.001
**Ground resistance R**_**g**_	Ohms (Ω)	0.01
**Snubber resistance R**_**s**_	Ohms (Ω)	1.0 × 10^−6^
**Fault capacitance Cs**	F	infinite
**Switching time**	seconds	0.2

Fig. 3 depicts the article's three-phase, 330 kV transmission line model, which was created and installed. It comprises of a 500-km-long Nigerian 330-kV transmission line that was simulated in Matlab/Simulink, as illustrated in Fig. 4. The ground resistance chosen is 0.01 based on the IEEE ground resistance standard of 0–50 for the ideal circumstance [43]. Table 1, Table 2 illustrate the input data for modelling a 500 km, 330 kV, 50 Hz transmission line. Simulations were done by placing the fault into the line 300 km distant. The parameters were selected with great care in compliance with the IEC 60909 standard [44].

In addition, the fault line minimum value of 0.001 Ω and the incipient fault angle (0° to 30°) were utilised to compute the maximum arc resistance value. Because a larger resistance value might create overvoltage and current, the system may be unable to detect small problems. This is why a low ground fault resistance is used to identify transient faults. As a result, the greater the fault resistance, the lower the defect detection. A three-phase fault simulator is used to mimic the failure at a different position on the transmission line.

The model's parameters are shown in Table 1, where R_1_ and R_2_ are the phases 1 and 2's positive and negative sequence resistance, respectively. Phases 1, 2, and 3 correspond to the positive and negative sequence inductances L_1_, L_2_, and L_3_, respectively, while phases 1, 2, and 3 correspond to the positive and negative sequence voltages C_1_, C_2_, and C_3_, respectively. The zero resistance sequence, capacitance, and inductance, respectively, are represented by R_0_, L_0_, and C_0_..

Twelve fault conditions were taken into consideration, and fault voltage and current data were acquired in a separate scenario. As shown in Table 3, they are a-g, b-g, c-g, a-b, b-c, a-c, a-b-g, b-c-g, a-c-g, a-b-c, a-b-c-g, and no-fault. Where a = fault at phase A, b = fault at phase B, c = fault at phase C, and g = the ground fault.

**Table 3:** Fault types in binary representation.

**Class**	**Fault type**	**L1 (a)**	**L2 (b)**	**L3 (c)**	**G (g)**
**1**	a-g	1	0	0	1
**2**	b-g	0	1	0	1
**3**	c-g	0	0	1	1
**4**	a-b	1	1	0	0
**5**	a-c	1	0	1	0
**6**	b-c	0	1	1	0
**7**	a-b-g	1	1	0	1
**8**	b-c-g	0	1	1	1
**9**	a-c-g	1	0	1	1
**10**	a-b-c	1	1	1	0
**11**	a-b-c-g	1	1	1	1
**12**	No fault	0	0	0	0

In the binary representation, the fault and no-fault states are represented by 1 and 0, respectively. It displays the fault number allocated to each fault circumstance.

## 4 Methodology

**Fig. 5:**
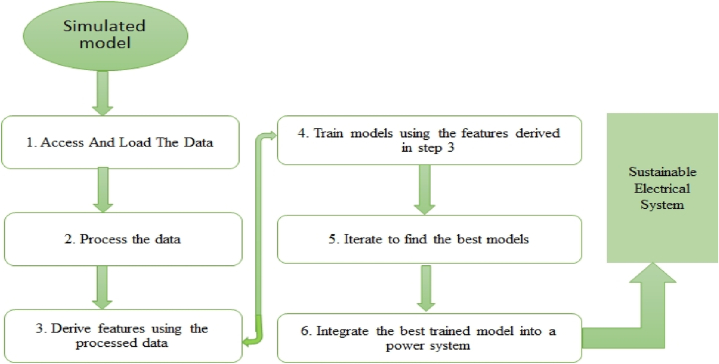
The data processing model for machine learning.

To train for accuracy, precision, and speed, phase and zero-sequence current fault and voltage data from simulated models may be utilised for fault classification, identification, and detection. The machine learning data processing model used for this paper is shown in Fig. 5. The simulated model's data must be obtained and put into the trainer. The data is then analyzed by searching for data points that are outside the fitting end of the remainder of the data to determine whether they may be ignored or considered [40].

The next step is to create features from the data by putting it into a machine-learning algorithm in order to improve model performance, model accuracy, model interpretability, and prevent over-drafting. A confusion matrix will be shown to compare the design classification with the actual data obtained. This is done before to constructing and training the model. The next step is to improve the model by deleting variables that are not associated, as shown by the correlation matrix. The fault data type was created by a 500 km, 500 kV, 50 Hz transmission line, and the dataset is divided into three categories: training data, testing data, and validation data. Each dataset will be trained before being evaluated for ultimate performance, accuracy, and error validation.

## 5 Data preparation and extraction

The faulty data were extracted using a Simulink model from Fig. 4, and the waveforms are generated from the model to show the frequency of fault occurrence. The data was normalised to boost the speed of training on the feature on a future basis. The rows and columns were normalised according to the formula in equation (8)

(8) $$x_{i}=\frac{\left(x_{i}-m_{x}^{\left(1\right)}\right)}{\sigma \;x}$$

Where $m_{x}^{\left(1\right)}$ represent the mean value of the row vector X, and σx is the standard deviation of row vector X. 1. The dataset consists of a 6 × 3336 matrix. The dataset is divided into three sections, with 23,336 fault data used for training, 5000 for testing and 5000 for validation.

**Fig. 6:**
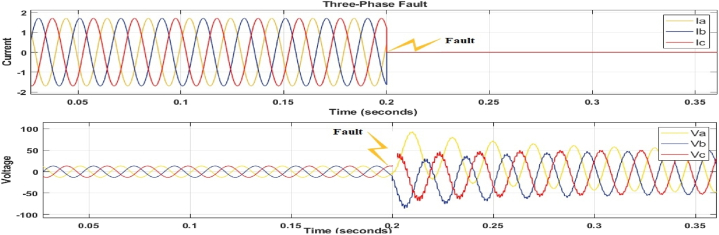
Three-phase to ground fault (a-b-c-g).

**Fig. 7:**
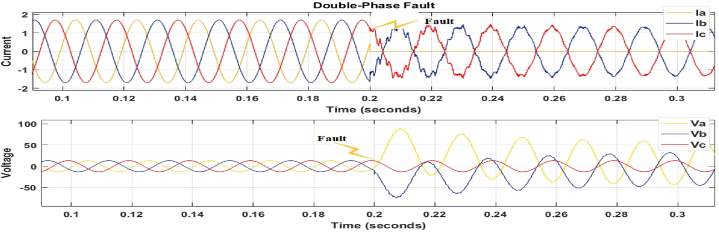
Double phase to ground Fault (a-b-g).

**Fig. 8:**
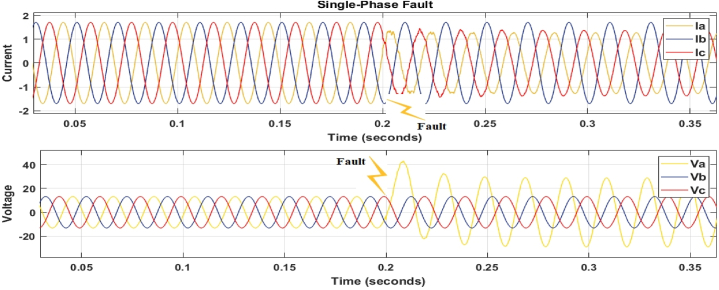
Single-phase to ground fault (a–g).

The graphs in Fig. 6, Fig. 7, Fig. 8 show the waveform to validate the presence of a fault in the network. The faulty current and voltage are generated and used for machine language training to classify and locate faults in the transmission line.

When a fault occurs, the power transmission line's fault current becomes abnormally high, while the fault voltage falls to a low level [45].

Fig. 6 shows the current and voltage waveforms of phases Va, Vb, Vc and Ia, Ib, Ic twisted owing to a three-phase to ground fault, with their magnitudes quickly falling. Fig. 6, Fig. 7 also show distorted voltage and current for phases B, C, and A owing to line failures. As a consequence of the issue, all of these waveforms show distortion; the fault model's switching time was set to 0.2 s, and the fault was placed 250 km from the transmission line.

Fig. 6, Fig. 7, Fig. 8 depict fault detection in four distinct fault types: no fault, single phase to ground fault, double line to ground fault, and three-phase to ground fault. Using the fault current and voltage data obtained, machine learning was used to train the data to identify, categorise, and pinpoint the transmission line issue. Fig. 6, Fig. 7, Fig. 8 indicate a significant rise in current, while Fig. 6 shows a voltage drop to zero, confirming the transmission line failure.

Fig. 6, Fig. 7, Fig. 8 confirm the occurrence of transmission line problems.

**Fig. 9:**
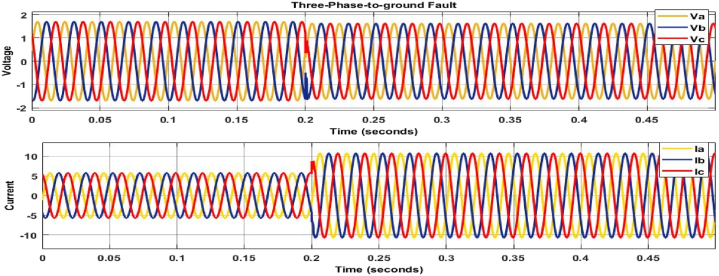
Three-phase-to-ground fault at 100 Ω fault resistance.

While selecting 0.001 as the minimal fault resistance to allow the model to identify transient faults. Raising the fault resistance reduces fault detection and may result in incorrect data that affects electrical installations, leading to system collapse, as seen in Fig. 9 where the voltage signal stays constant but the current signal increases.

## 6 Result and discussion

The ANN technique is used to classify and detect the fault in the network, and a data size of 33,336 was generated from Fig. 4 and used for the algorithm's training. The data size was chosen base on the simulated faults result of the voltage and current waveform. The data is divided into training, testing and validation in 80%, 15% and 5%, respectively to avoid over-fitting. Phase value of faulty current and voltage was used as input data, while the three-phase value and ground phase were used as the input layer. The sigmoid function is used as the activation function, given in equation (9) below.

(9) $$f\left(x\right)=\frac{1}{1+e^{-x}}$$

Where x is the net input chosen through the trial and error method till the training is fitted.

The selection of the sigmoid activation function was based on the target input value of 0 and 1 and due to the normalisation of the dataset in Table 4 though, the tanH activation function was als used but the accuracy was the same with time of execution was 0.006 s. The activation function helps to maintain the output or predicted value in a particular range with high accuracy and efficiency of the model.

**Table 4:** Fault location at different distances.

Fault Type	Actual Fault Distance (km)	Estimated Fault Location (km)	% Error
Single Line to Ground	100	103.78	3.78
	250	252.65	1.06
	450	448.67	−0.30
Double line to Ground	100	101.67	1.67
	250	253.98	1.59
	450	452.10	0.47
Line To Line	100	100.98	0.98
	250	252.77	1.11
	450	450.98	0.22
Three Phase To Ground	100	100.87	0.87
	250	252.10	0.84
	450	451.98	0.44

The percentage error between the estimated distance and the actual distance is noted to be very low confirming the viability of the model.

A total of six input parameters, 15 input layers, 2 output layers and 4 output result (L-G, L-L, L-L-G, and L-L-L-G) is used for the training. The algorithm performance was analyzed, and the Mean Square Error (MSE), epoch and training time was also considered.

A confusion matrix was used to evaluate the model's performance, and it compares the actual target values with those predicted by the ANN model. It also gives a holistic view of the model's performance and the type of error involved in the system. The result also explains the practical use of ANN in transmission line faults management system plan.

**Fig. 10:**
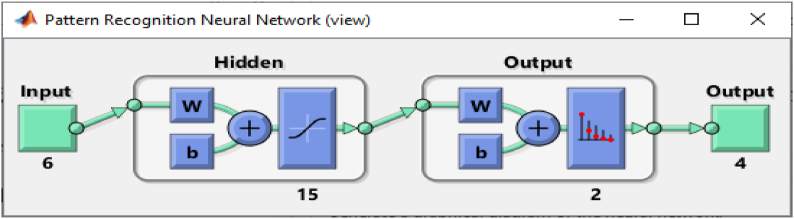
ANN Training configuration for fault Detection and classification.

Fig. 10 shows the configuration used for the training, which consists of a 6-15-2-4 structure. This configuration was selected after a training series, and the best data fitting was achieved, and the hidden layer was set at 15 to achieve the best configuration. This consists of 6 input data (three-phase current and voltage I_a_, I_b_, I_c_ and V_a_, V_b_, V_c_) and four output data (L-G, L-L, L-L-G, L-L-L/L-L-L-G) is same as a-g, b-c-g and a-b-c-g, which is used for the training.

**Fig. 11:**
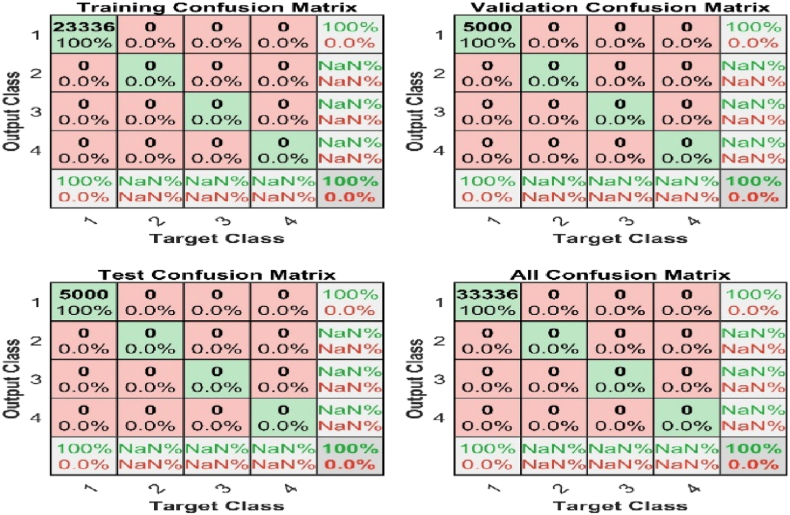
ANN Confusion Matrix for fault detection.

The confusion matrix in Fig. 11 summarises the model's fault detection prediction. It tests the dataset or validates data with expected values and makes predictions in each row in the dataset. It also makes correct predictions of each class and the number of incorrect predictions. The no-fault condition represents 1, the single line to ground fault represents 2, the double line to ground represent 3, and the three phases to ground fault represent 4. About 33,336 fault data was used for training with 100% accuracy and 0% confusion state. Meanwhile, 23,336 datasets were used to validate and train the fault data with 100% accuracy, as seen in Fig. 11.

**Fig. 12:**
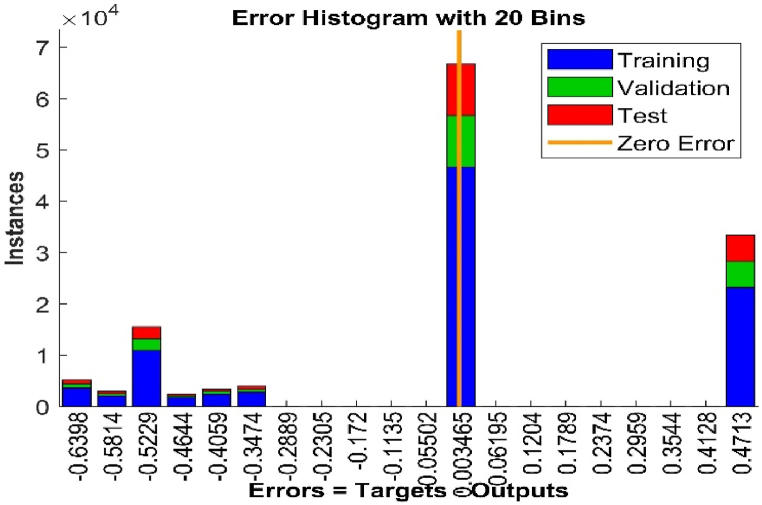
Error histogram for the ANN network.

The error histogram in Fig. 12 shows the error between the target and predicted values after the ANN network has been trained. The difference between the target and the output has the corresponding zero error value of −0.00739, and it shows that the error is minimal.

**Fig. 13:**
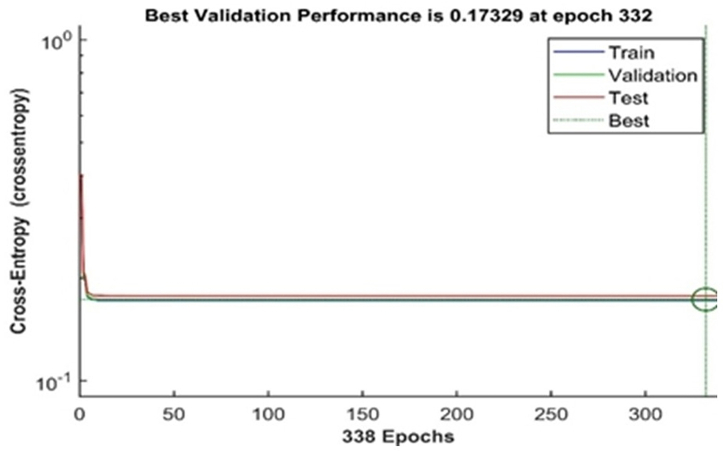
ANN network performance.

The network's best validation performance was 0.17329 at 332 epochs, as shown in Fig. 13. This is a good performance because the network is fitted, and the best fit line is close to the train line, validation and test line because they have related features that do effective training, as shown in Fig. 13 above.

**Fig. 14:**
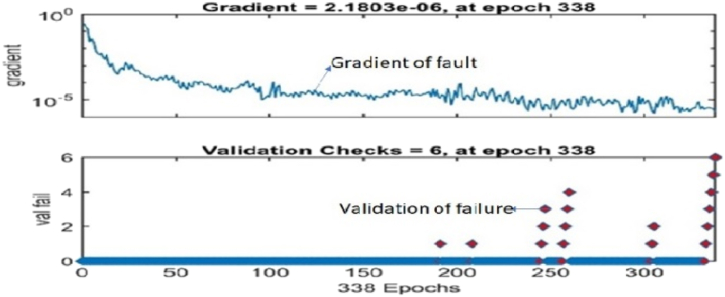
Validation failures of faults.

From Fig. 14, the model has a maximum level of allowable failure of 6 at 338 epochs and a gradient of 2.1803 × 10^−6^ This graph shows that the model is good because the network gradient performance is in perfect condition and minimal due to its performance.

### 6.1 Fault localisation result

**Fig. 15:**
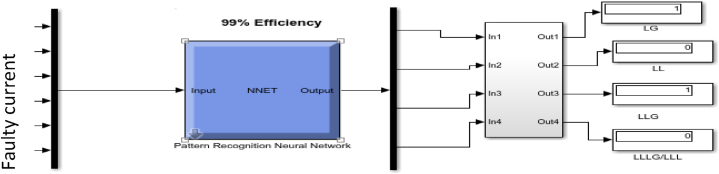
L–L and three-phase fault detected at 200 km distance.

**Fig. 16:**
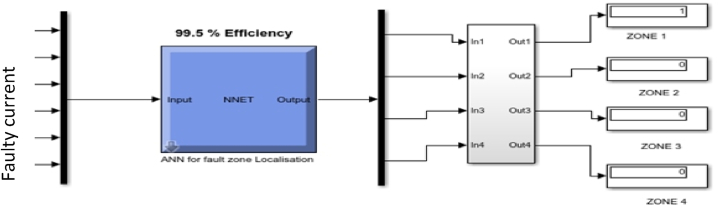
Fault location at three zones.

**Fig. 17:**
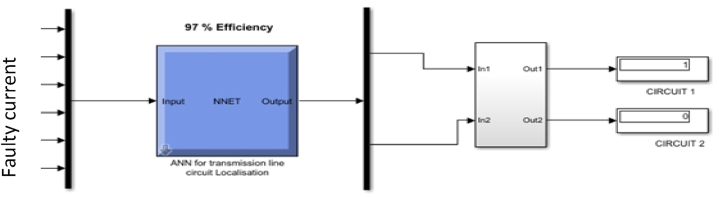
Fault location at line 2.

The model is also designed for fault localisation in the transmission line using Simulink to generate a neural network classifier to predict the fault location. The input parameters are the fault current and voltage of a three-phase line. The input is then fed into the classifier to detect the fault location. These fault types and fault locations are displayed on a screen for easy accessibility. Possible action is taken for a fast and effective fault management plan, as seen in Fig. 15, Fig. 16, Fig. 17.

In Fig. 15, the line-to-line fault and the three-phase-to-ground fault were detected at the 200 km distance of the transmission line.

Also, Fig. 16 shows that fault was in zone 2,3 and 4 while zone 1 had no fault. This method clearly shows the type of fault detected and the zone where the fault is found. The network is segmented into zones and circuits for easy identification, as seen in Fig. 17, which shows that the fault was situated in the circuit or line 2. This can be replicated to other fault conditions to detect and locate the exact spot of the fault for easy isolation and restoration of power.

This model is superior to others because it clearly states the type of fault and the location of the fault at any given time. This will assist the maintenance team to quickly clearing the fault and is helpful in the transmission line fault management system.

Table 4 shows the actual distance and the estimated distance of fault location at different points in the transmission line.

### 6.2 Comparative analysis of the ANN technique with respect to latency and other machine learning techniques

Rapid and timely identification of faults aids in separating the damaged line, protecting the system from the dangerous repercussions of the faults. The adverse effect may lead to power outages, damage to installation, cost implications, thereby affecting the economy. Furthermore, information from fault categorisation can enhance the prompt discovery of a faulty point, minimising the time necessary to remove faults and swiftly restore electricity service. As a result, many scientific investigations are being conducted to develop a robust, accurate, rapid and timely strategy for the detection and localisation of the faults on transmission lines [46].

Table 5 compares the execution timings of multiple fault localisation and detection methods in the transmission line. In comparison, WPT and ANN take 110 s, WT takes 4.000 s, PMU takes 0.800 s, while the ANN technique takes 0.0017 s to execute. Because it exceeds the competition in terms of speed with a percentage error of 0.007%, the ANN is preferred for defect localisation and detection.

**Table 5:** Comparison of percentage error and speed of execution of different algorithms used for fault localisation.

Algorithm used	Input used	Percentage Error	Time of execution
WPT and ANN [47]	Current signal	2.05%	110 s
Adaptive Network-Based Fuzzy Inference System (ANFIS) [48]	Three-phase current	0.07%	2.6 s
Radial Bias Function (RBF) [49]	Positive sequence voltage and current waveform	2.8%	0.53 s
Discrete WT [50]	Single-phase voltage signal	1.67%	4.0 s
Linear discrimination principle [51]	Three phases current and voltage signal	2.66%	0.032 s
PMU [5]	Voltage signal	4%	0.8 s
DWT & ANN [52]	Current signal	12.78% for ANN and 0% for DWT	0.15 s
Proposed ANN Solution	Voltage and current signal	0.007%	0.0017 s

For effective fault detection and localisation technique to be executed, there must be a quick response to fault.

Noise signals reduce the accuracy of identifying and detecting transmission line defects. Noise signals such as voltage sag, transient, harmonics, and voltage interruption must be considered while selecting and extracting fault characteristics. The DWT was employed for feature extraction and the SVM was used for fault classification in Ref. [53], with an accuracy of 100% when there is no disturbance and 98% and 95.6% accuracy at 30 dB and 20 dB noise, respectively.

**Fig. 18:**
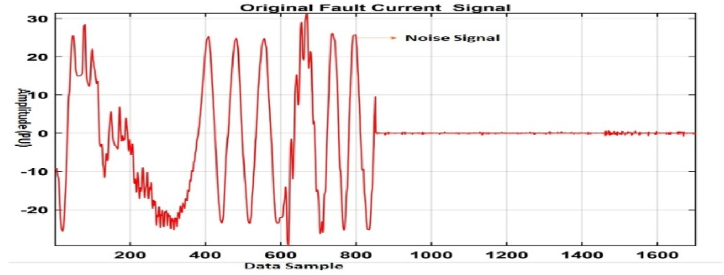
Fault signal with noise.

**Fig. 19:**
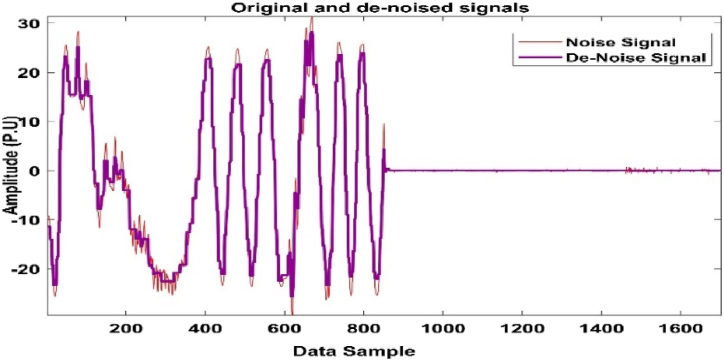
Fault with De-noise signal.

Fig. 18, Fig. 19 depict the fault signal with noise, whereas Fig. 19 depicts the de-noised signal.

To get a de-noise signal, it is recommended that noise be removed using the DWT approach during fault extraction. The data gathered from this approach will be employed in an ANN classifier for extremely accurate results.

**Fig. 20:**
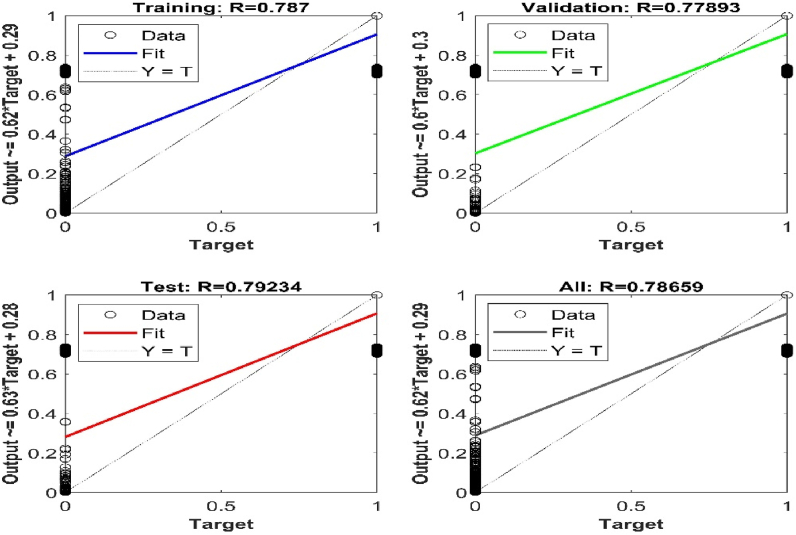
Regression Fit for the noise signal data.

Fig. 18, Fig. 19 show a de-noise signal and a faulty signal with noise, respectively. The model was examined for noise regularisation by adding noise to the input vector during training and using the tanH activation function to train the noise vector, and the accuracy decreased substantially to 78%, as shown in Fig. 20.

## 7 Conclusion

This paper has investigated the use of artificial neural networks as a technique for fault detection and localisation in the transmission line. A 330 kV, 500 km, 50 Hz three-phase transmission line was modelled using Matlab/Simulink to generate the RMS value of faulty voltage and current signals from the defective line. About 12 different fault scenarios were considered, and 33,336 data samples of faulty current and voltage were taken from various locations in the transmission line to detect faults using ANN and to use the module for fault localisation. The different faults were discussed, especially single, double, and three-phase. The data were trained for accuracy, speed and precision, with an accuracy of 100\% for fault detection and 99\% fault localisation at separate locations. The time for fault detection is important in fault protection and this paper has focused on the speed of execution for prompt detection of fault. This technique produces excellent results compared to other conventional methods like the SVM and DWT.

However, the ANN technique have some drawbacks like large volume of data are required for optimal performance and difficulty in determining the proper network structure for best performance of the model. This paper has some limitations which include: The fault data generated were only based on the minimum fault resistance of 0.001 $\Omega$ and the fault angle can be varied by increasing or decreasing it for optimal fault detection. Also, Noise was not taken into account during simulation though it was mentioned.

In practice, The results generated from this can also be recommended as a basis for the design of effective fault management and protection of power systems.

## Author contribution statement

Vincent Nsed Ogar: Conceived and designed the experiments; Performed the experiments; Analyzed and interpreted the data; Contributed reagents, materials, analysis tools or data; Wrote the paper.

Sajjad Hussain: Analyzed and interpreted the data; Contributed reagents, materials, analysis tools or data; Wrote the paper.

Kelum A.A. Gamage: Conceived and designed the experiments; Analyzed and interpreted the data; Contributed reagents, materials, analysis tools or data; Wrote the paper.

## Funding statement

This research did not receive any specific grant from funding agencies in the public, commercial, or not-for-profit sectors.

## Data availability statement

No data was used for the research described in the article.

## Declaration of interest’s statement

The authors declare no conflict of interest.

## References

[1] Singh M.R. (2015). Fault Classification in electric power transmission lines using support vector machine. Int. J. Innov. Res. Sci. Technol.

[2] Ul Haq E., Jianjun H., Li K., Ahmad F., Banjerdpongchai D., Zhang T. (2021). Improved performance of detection and classification of 3-phase transmission line faults based on discrete wavelet transform and double-channel extreme learning machine. Electr. Eng..

[3] Rahmati A., Adhami R. (2014). A fault detection and classification technique based on sequential components. IEEE Trans. Ind. Appl..

[4] Chen K., Huang C., He J. (2016). Fault detection, classification and location for transmission lines and distribution systems: a review on the methods. High Volt..

[5] Gopakumar P., Reddy M.J.B., Mohanta D.K. (2015). Transmission line fault detection and localisation methodology using PMU measurements. IET Gener., Transm. Distrib..

[6] Swetapadma A., Yadav A. (2016). Directional relaying using support vector machine for double circuit transmission lines including cross-country and inter-circuit faults. Int. J. Electr. Power Energy Syst..

[7] Xu Z.Y. (2008). A distance protection relay for a 1000-kV UHV transmission line. IEEE Trans. Power Deliv..

[8] Rui G., Feng Z., Lei C., Jun Y. (2014). A mobile robot for inspection of overhead transmission lines. Proceed. 3rd Int. Conf. Appl. Robot. Power Ind. CARPI.

[9] Katrašnik J., Pernuš F., Likar B. (2010). A survey of mobile robots for distribution power line inspection. IEEE Trans. Power Deliv..

[10] Sawada J., Kusumoto K., Maikawa Y., Munakata T., Ishikawa Y. (1991). A mobile robot for inspection of power transmission lines. IEEE Trans. Power Deliv..

[11] Montambault S., Pouliot N. (2010).

[12] Youssef O.A.S. (2004). Combined fuzzy-logic wavelet-based fault classification technique for power system relaying. IEEE Trans. Power Deliv..

[13] Jayabharata Reddy M., Mohanta D.K. (2007). A wavelet-fuzzy combined approach for classification and location of transmission line faults. Int. J. Electr. Power Energy Syst..

[14] Wang H., Keerthipala W.W.L. (1998). Fuzzy-neuro approach to fault classification for transmission line protection. IEEE Trans. Power Deliv..

[15] Prasad A., Edward J.B. (2016). Application of wavelet technique for Fault Classification in transmission systems. Procedia Comput. Sci..

[16] Babu K., Tripathy M., Singh A. (2011). Recent techniques used in transmission line protection: a review. Int. J. Eng. Sci. Technol..

[17] Hossam-Eldin A., Lotfy A., Elgamal M., Ebeed M. (2016). EEEIC 2016 - International Conference on Environment and Electrical Engineering.

[18] Swetapadma A., Yadav A. (2018). An artificial neural network-based solution to locate the multilocation faults in double circuit series capacitor compensated transmission lines. Int. Transact. Elect. Energy Sys..

[19] Uzubi U., Ekwue A., Ejiogu E. (2017). Proceedings - 2017 IEEE PES-IAS PowerAfrica Conference: Harnessing Energy, Information and Communications Technology (ICT) for Affordable Electrification of Africa, PowerAfrica 2017.

[20] dos Santos R.C., Senger E.C. (2011). Transmission lines distance protection using artificial neural networks. Int. J. Electr. Power Energy Syst..

[21] Prasad A., Edward J.B. (2017). Proceedings of 2017 11th International Conference on Intelligent Systems and Control, ISCO 2017.

[22] Osofisan P.B., Nwaeke C.N. (2010). Application of artificial neural network (ANN) for short-term load forecasting (case study on national control centre (PHCN) oshogbo, osun state, Nigeria). J. Eng. Appl. Sci..

[23] Chang G.W., Hong Y.H., Li G.Y. (2019). A hybrid intelligent approach for classification of incipient faults in transmission network. IEEE Trans. Power Deliv..

[24] Jaya Bharata Reddy M., Gopakumar P., Mohanta D.K. (2016). A novel transmission line protection using DOST and SVM. Eng. Sci. Tech. Int. J..

[26] Costa F.B., Silva K.M., Souza B.A., Dantas K.M.C., Brito N.S.D. (2006). IEEE International Conference on Neural Networks - Conference Proceedings.

[27] Tayeb E.B.M., Rhim O.A.A.A. (2012). Proceedings of the 2011 International Conference and Utility Exhibition on Power and Energy Systems: Issues and Prospects for Asia, ICUE 2011.

[28] Roy N., Bhattacharya K. (2015). Detection, classification, and estimation of fault location on an overhead transmission line using s-transform and neural network. Elec. Power Compon. Syst..

[29] Abdulwahid A.H. (2019). 2019 2nd International Conference of the IEEE Nigeria Computer Chapter, Nigeria Comput Conf 2019.

[30] Mishra M., Rout P.K. (2018). Detection and classification of micro-grid faults based on HHT and machine learning techniques. IET Gener., Transm. Distrib..

[31] Chopra P., Yadav S.K. (2016). 2015 IEEE Recent Advances in Intelligent Computational Systems, RAICS 2015.

[32] Mukherjee A., Kundu P.K., Das A. (2021). A supervised principal component analysis-based approach of fault localisation in transmission lines for single line to ground faults. Electr. Eng..

[33] Rai P., Londhe N.D., Raj R. (2021). Fault classification in power system distribution network integrated with distributed generators using CNN. Elec. Power Syst. Res..

[34] Elnozahy A., Sayed K., Bahyeldin M. (2019). IEEE Conference on Power Electronics and Renewable Energy, CPERE 2019.

[35] Netsanet S., Zhang J., Zheng D. (2018). Bagged decision trees based scheme of microgrid protection using windowed fast fourier and wavelet transforms. Electronics (Switzerland).

[36] Mishra D.P., Samantaray S.R., Joos G. (2016). A combined wavelet and data-mining based intelligent protection scheme for microgrid. IEEE Trans. Smart Grid.

[37] Ogar V.N., Gamage K.A.A., Hussain S. (2022). Protection for 330 kV transmission line and recommendation for Nigerian transmission system: a review. Int. J. Electr. Comput. Eng..

[38] Kar S., Samantaray S.R. (2014). Time-frequency transform-based differential scheme for microgrid protection. IET Gener., Transm. Distrib..

[39] Godse R., Bhat S. (2020). Mathematical morphology-based feature-extraction technique for detection and classification of faults on power transmission line. IEEE Access.

[40] Raza A., Benrabah A., Alquthami T., Akmal M. (2020). A review of fault diagnosing methods in power transmission systems. Appl. Sci..

[41] da Silva I.N., Spatti D.H., Flauzino R.A., Liboni L.H.B., dos Reis Alves S.F. (2016).

[42] Xiang W., Tran H.D., Johnson T.T. (2018). Output reachable set estimation and verification for multilayer neural networks. IEEE Transact. Neural Networks Learn. Syst..

[43] de Andrade V., Sorrentino E. (2011). 2010 IEEE/PES Transmission and Distribution Conference and Exposition: Latin America, T and D-LA 2010.

[44] Sweeting D. (2012). Applying IEC 60909, fault current calculations. IEEE Trans. Ind. Appl..

[45] Fei Chunguo, Qin Junjie (2021). Fault Location after Fault Classification in transmission line using voltage amplitudes and support vector machine. Russ. Electr. Eng..

[46] Moradzadeh A., Teimourzadeh H., Mohammadi-Ivatloo B., Pourhossein K. (2022). Hybrid CNN-LSTM approaches for identification of type and locations of transmission line faults. Int. J. Electr. Power Energy Syst..

[47] Ekici S., Yildirim S., Poyraz M. (2008). Energy and entropy-based feature extraction for locating fault on transmission lines by using neural network and wavelet packet decomposition. Expert Syst. Appl..

[48] Sadeh J., Afradi H. (2009). A new and accurate fault location algorithm for combined transmission lines using Adaptive Network-Based Fuzzy Inference System. Elec. Power Syst. Res..

[49] Gayathri K., Kumarappan N. (2010). Accurate fault location on EHV lines using both RBF based support vector machine and SCALCG based neural network. Expert Syst. Appl..

[50] Mahanty R., Gupta P. (2004). Voltage stability analysis in unbalanced power systems by optimal power flow. IEE Proceed. Generat. Trans..

[51] Zhang Y.-G., Wang Z.-P., Zhang J.-F., Ma J. (2011). Fault localisation in electrical power systems: a pattern recognition approach. Int. J. Electr. Power Energy Syst..

[52] Ray P., Mishra D.P., Dey K., Mishra P. (2018). Fault Detection and classification of a transmission line using discrete wavelet transform & artificial neural network. Proceed. - 2017 Int. Conf. Info. Tech., ICIT 2017.

[53] Erişti H., Uçar A., Demir Y. (2010). Wavelet-based feature extraction and selection for classification of power system disturbances using support vector machines. Elec. Power Syst. Res..

